# 4S‐AF scheme and ABC pathway guided management improves outcomes in atrial fibrillation patients

**DOI:** 10.1111/eci.13751

**Published:** 2022-02-01

**Authors:** Yutao Guo, Jacopo F. Imberti, Agnieszka Kotalczyk, Yutang Wang, Gregory Y. H. Lip, the ChiOTEAF Registry Investigators

**Affiliations:** ^1^ Department of Pulmonary Vessel and Thrombotic Disease Sixth Medical Centre Chinese PLA General Hospital Beijing China; ^2^ Liverpool Centre for Cardiovascular Science University of Liverpool and Liverpool Heart & Chest Hospital Liverpool UK; ^3^ Cardiology Division Department of Biomedical, Metabolic and Neural Sciences University of Modena and Reggio Emilia, Policlinico di Modena Modena Italy; ^4^ Department of Cardiology, Congenital Heart Diseases and Electrotherapy Silesian Centre for Heart Diseases Medical University of Silesia Zabrze Poland; ^5^ Department of Cardiology Second Medical Centre Chinese PLA General Hospital Beijing China; ^6^ Aalborg Thrombosis Research Unit Department of Clinical Medicine Aalborg University Aalborg Denmark

**Keywords:** 4S‐AF scheme, ABC pathway, atrial fibrillation, guidelines

## Abstract

**Background:**

The 4S‐AF scheme and the ABC pathway for integrated care have been proposed to better characterize and treat patients with atrial fibrillation (AF). We aimed to evaluate the assessment of the 4S‐AF scheme and ABC pathway in Chinese AF patients.

**Methods:**

The ChiOTEAF is a prospective, observational, multicentre registry. Consecutive AF patients from 44 centres across 20 Chinese provinces with available 1‐year follow‐up data were included.

**Results:**

A total of 6419 patients were included, median age 76 years (interquartile range 67–83; 39.1% female). Of these, 3503 (59.8%) patients were not characterized using the 4S‐AF scheme and not management according to the ABC pathway (group 1); 1795 (28.0%) were characterized according to the 4S‐AF scheme but ABC pathway non‐adherent or vice versa (group 2); and 1121 (17.4%) characterized according to the 4S‐AF scheme and were ABC pathway adherent (group 3).

As compared with group 1, group 2 and group 3 were independently associated with lower odds of the composite endpoint of all‐cause death/any thromboembolic event, with the greatest benefit observed in group 3 (OR: 0.19; 95% CI 0.12–0.31) [for group 2: OR: 0.28; 95% CI 0.20–0.37]. Similar results were observed for all‐cause death (group 2: OR: 0.18; 95% CI 0.12–0.27; group 3: OR: 0.14; 95% CI 0.07–0.25).

**Conclusions:**

In a contemporary real‐word cohort of Chinese AF patients, it is feasible to characterize and manage AF patients using the novel 4S‐AF scheme and ABC pathway for integrated care. The use of both these tools is associated with improved clinical outcomes.

## 1 INTRODUCTION

Atrial fibrillation (AF) is a complex and multifaceted disease, often associated with several cardiovascular and non‐cardiovascular risk factors and comorbidities,^1^, ^2^ posing significant challenges to its comprehensive characterization.

Different AF classifications have been proposed over time,^3^, ^4^, ^5^ but most of them addressed only single domains of AF or patient‐related characteristics (temporal pattern, symptom severity or underlying comorbidity), thus potentially limiting the delivery of holistic care. To overcome this issue, the latest European Society of Cardiology guidelines for the diagnosis and management of AF^3^ proposed a paradigm shift towards a more structured characterization (rather than classification) of AF, addressing 4 domains (Stroke risk, Symptom severity, Severity of AF burden, Substrate) aiming to streamline the assessment of AF patients at all healthcare levels and facilitating treatment decision‐making (4S‐AF scheme).^6^

Following AF characterization, the ABC (Atrial fibrillation Better Care) pathway^7^ has been proposed as a simple ‘step‐by‐step’ multidisciplinary approach to holistic care for AF patients. ‘A’ refers to Avoid stroke with Anticoagulation, ‘B’ to Better symptom management with patient‐centred, symptom directed decisions on rate or rhythm control, and ‘C’ to Comorbidity and Cardiovascular risk factor management, including lifestyle changes. The impact of this approach on patients’ outcome is well‐recognized^8^, ^9^, ^10^, ^11^, ^12^ and also highlights the role of addressing multimorbidity on patients’ prognosis. Indeed, only 1 in 10 of deaths associated with AF are due to stroke, while ≥7 in 10 are due to cardiovascular causes.^13^, ^14^ However, data on the clinical utility of such a guideline‐directed management for AF patients, comprised of clinical characterization through the novel 4S‐AF scheme and ABC pathway adherent approach to integrated care, are limited.

Given that, we investigated the assessment of the 4S‐AF scheme and the ABC pathway in the nationwide Optimal Thromboprophylaxis in Elderly Chinese Patients with Atrial Fibrillation (ChiOTEAF) registry and second, we evaluated the potential prognostic implications of such a guideline‐recommended approach.

## 2 METHODS

### 2.1 Study design and population

The ChiOTEAF registry is a prospective, observational, large‐scale multicentre study conducted between October 2014 and December 2018 in 44 sites from 20 provinces in China. Detailed description of the study design has been previously published.^15^ In brief, consecutive patients with electrocardiographically documented clinical AF within 12 months prior to enrolment were included. Follow‐up was regularly performed up to 2 years after enrolment. Data were collected at enrolment and follow‐up visits by local investigators. The ChiOTEAF registry was approved by the Central Medical Ethics Committee of Chinese PLA General Hospital, Beijing, China (approval no S2014‐065‐01), and local institutional review boards.

### 2.2 Data collection and study outcomes

Data on demographics and comorbidities were collected at baseline. Variables included in the registry and their definitions were designed to match the EURObservational Research Programme Atrial Fibrillation (EORP‐AF) Long‐term General Registry.^16^ The CHA_2_DS_2_‐VASc score,^17^ HAS‐BLED score^18^ and European Heart Rhythm Association (EHRA) symptom score^19^ were determined as previously described. AF classification and pattern was based on the European Society of Cardiology guidelines for temporal classification of AF.^20^, ^21^ Left atrial (LA) enlargement was defined as LA diameter over 40 mm.

We retrospectively evaluated the ABC pathway at baseline visit according with its original definition.^7^ The *‘A’*‐ *domain* referred to proper oral anticoagulant (OAC) treatment according to patients’ thromboembolic (TE) risk, the *‘B’*‐ *domain* referred to actual AF symptoms control and the *‘C’*‐ *domain* referred to disease‑specific treatment of comorbidities according to current guidelines, or no management in case of no comorbidities. The definition and interpretation of each domain is provided in Table S1. Patients were considered ABC pathway adherent when compliant with all the three domains.

The 4S‐AF classification scheme was retrospectively evaluated in its four original domains^6^: stroke risk (*St*), symptoms severity (*Sy*), severity of AF burden (*Sb*) and substrate (*Su*). Detailed definition each domain is provided in Table S2. The sum of each individual domain was used to calculate a 4S‐AF score, with a maximum of 9 (*St* = 1, *Sy* = 2, *Sb* = 2, *Su* = 4).

For the purpose of this analysis, we divided the cohort into 3 groups. Group 1 included patients not characterized using the 4S‐AF scheme and not management according to the ABC pathway; group 2 included patients who were optimally characterized according to the 4S‐AF scheme but were ABC pathway non‐adherent or not characterized according to the 4S‐AF scheme and ABC pathway adherent; and group 3 included patients who were optimally characterized according to the 4S‐AF scheme and were ABC pathway adherent. The outcomes of interest were all‐cause mortality, any TE event, and major bleeding at 1‐year follow‐up. Patients without outcome data at 1‐year follow‐up were excluded from this analysis.

### 2.3 Statistical analysis

Categorical variables were expressed as counts and percentages and continuous variables as median and interquartile range (IQR). Between‐group comparisons were made by using a chi‐square test for categorical variables and Student's *t* test or median test for continuous variables. Multivariable regression analysis was performed for the outcomes of interest to test the effects of management(s) based on the 4S‐AF scheme and ABC pathway after adjustment for age, gender, chronic kidney disease, chronic obstructive pulmonary disease, coronary artery disease, diabetes mellitus, heart failure, hypercholesterolaemia, hypertension and prior stroke. Results were expressed as odds ratio (OR), 95% confidence interval (CI) and *p* value. In all analyses, a two‐sided *p* value <.05 was considered statistically significant. Statistical analyses were performed using spss ^®^ version 24 (IBM Corp).

## 3 RESULTS

**FIGURE 1:**
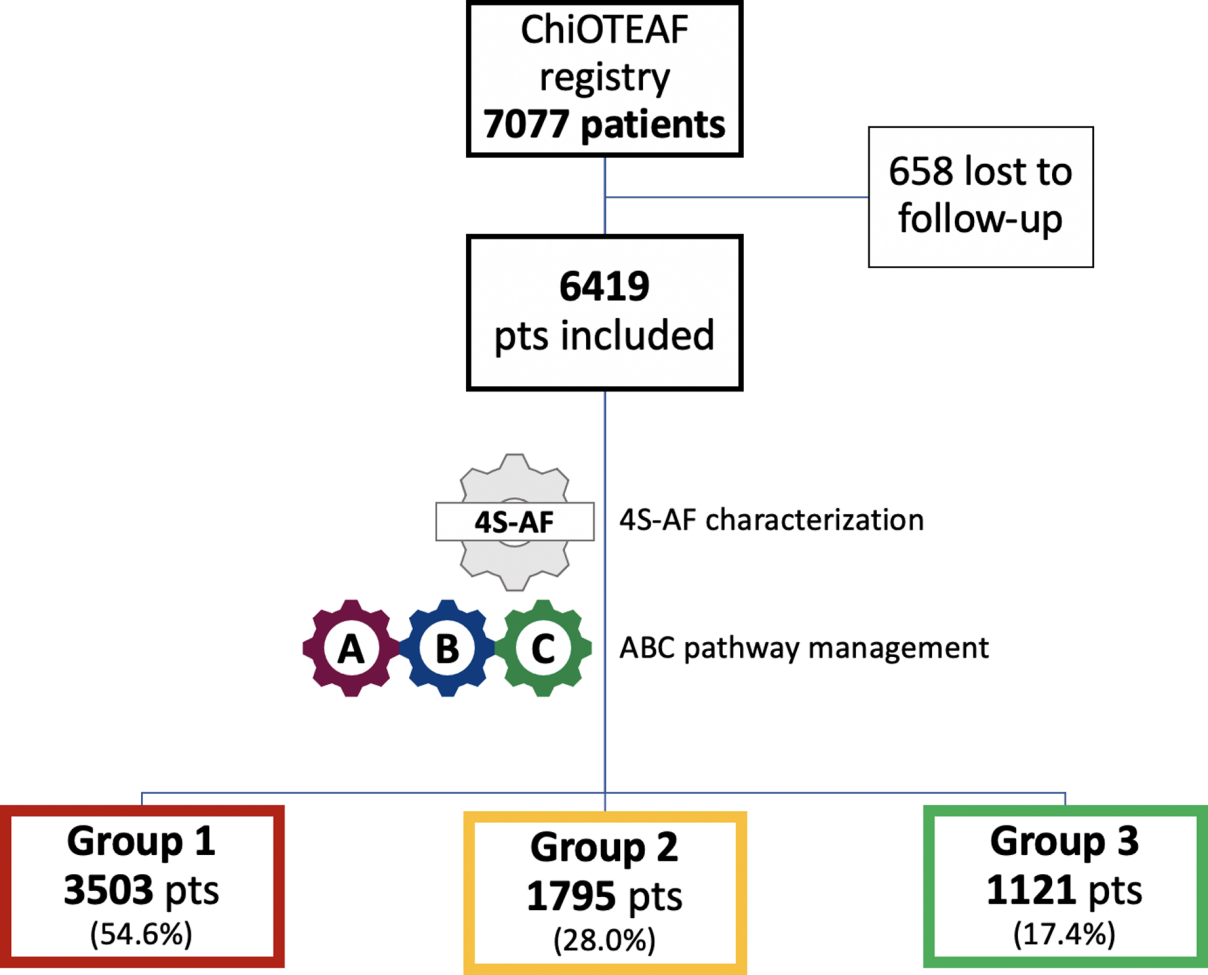
Flow chart of patients’ selection. Group 1 includes patients not characterized using the 4S‐AF scheme and not management according to the ABC pathway; group 2 includes patients characterized according to the 4S‐AF scheme but ABC pathway non‐adherent or vice versa; and group 3 includes patients characterized according to the 4S‐AF scheme and ABC pathway adherent. ABC, atrial fibrillation better care; Pts, patients

Out of the original cohort of 7077 patients, a total of 6419 (90.7%) patients were included in the present analysis. The median age of patients was 76 (IQR 67–83) years with 2511 (39.1%) females (Table 1). Median CHA_2_DS_2_‐VASc and HAS‐BLED scores were 3 (IQR 2–5) and 2 (IQR 1–3), respectively. Of these, 3503 (54.6%) patients were not characterized using the 4S‐AF scheme and not management according to the ABC pathway (group 1; guideline non‐adherent management); 1795 (28.0%) were optimally characterized according to the 4S‐AF scheme but were ABC pathway non‐adherent or vice versa (group 2; ie 4S‐AF scheme characterization, ABC pathway non‐adherence or vice versa); and 1121 (17.4%) optimally characterized according to the 4S‐AF scheme and were ABC pathway adherent (group 3; 4S‐AF scheme and ABC pathway adherent) (Figure 1). Patients in group 1 were older (*p *< .01) and female were less represented (*p *< .01). Patient baseline characteristics are summarized in Table 1, and patient treatments addressing anticoagulation, rate/rhythm control and comorbidities are reported in Table S3.

**TABLE 1:** Baseline characteristics of the study cohort

	Total *N* = 6419 *n* (%)	Group 1 *N* = 3503 *n* (%)	Group 2 *N* = 1795 *n* (%)	Group 3 *N* = 1121 *n* (%)	*p* Value
Age^a^ (*n* = 6415)	76 (67–83)	78 (69–84)	75 (65–81)	73 (64–80)	<.01
Female (*n *= 6415)	2511 (39.1)	1244 (35.6)	782 (43.6)	485 (43.3)	<.01
Diabetes mellitus (*n* = 6402)	1682 (26.3)	893 (25.6)	481 (26.8)	308 (27.5)	.39
Hypercholesterolaemia (*n* = 6312)	2800 (44.4)	1284 (37.7)	876 (49.2)	640 (57.1)	<.01
Heart failure (*n* = 6402)	2290 (35.8)	1316 (37.8)	635 (35.4)	339 (30.2)	<.01
Coronary artery disease (*n* = 6279)	3032 (48.3)	1624 (48.0)	920 (51.8)	488 (43.5)	<.01
Hypertension (*n* = 6402)	4072 (63.6)	2176 (62.4)	1094 (60.9)	802 (71.5)	<.01
COPD (*n* = 6403)	598 (9.3)	400 (11.5)	134 (7.5)	64 (5.7)	<.01
Prior ischemic stroke	1588 (24.7)	929 (26.5)	371 (20.7)	288 (25.7)	<.01
Chronic kidney disease	790 (12.3)	523 (14.9)	184 (10.3)	83 (7.4)	<.01
Prior major bleeding (*n* = 6403)	266 (4.2)	191 (5.5)	71 (4.0)	4 (0.4)	<.01
Clinical type of AF (*n* = 5499)					
First diagnosed AF	948 (17.2)	474 (18.0)	327 (18.7)	147 (13.1)	<.01
Paroxysmal AF	2460 (44.7)	1013 (38.5)	882 (50.4)	565 (50.4)	
Persistent AF	1016 (18.5)	408 (15.5)	322 (18.4)	286 (25.5)	
Long‐standing persistent AF	188 (3.4)	85 (3.2)	56 (3.2)	47 (4.2)	
Permanent AF	887 (16.1)	648 (24.7)	163 (9.3)	76 (6.8)	
LA diameter^a^ (*n* = 4457)	42 (37–47)	42 (38–48)	41 (36–46)	42 (38–47)	<.01
CHA_2_DS_2_VASc^a^ (*n* = 5925)	3 (2–5)	4 (2–5)	3 (2–4)	3 (2–5)	<.01
HAS‐BLED^a^ (*n* = 6031)	2 (1–3)	2 (1–3)	2 (1–3)	2 (1–3)	<.01
4S‐AF score^a^ (*n* = 2588)	4 (3–5)	–	4 (3–5)	5 (4–5)	<.01

Abbreviations: AF, atrial fibrillation; COPD, chronic obstructive pulmonary disease; LA, left atrial.

^a^ Median (interquartile range).

**FIGURE 2:**
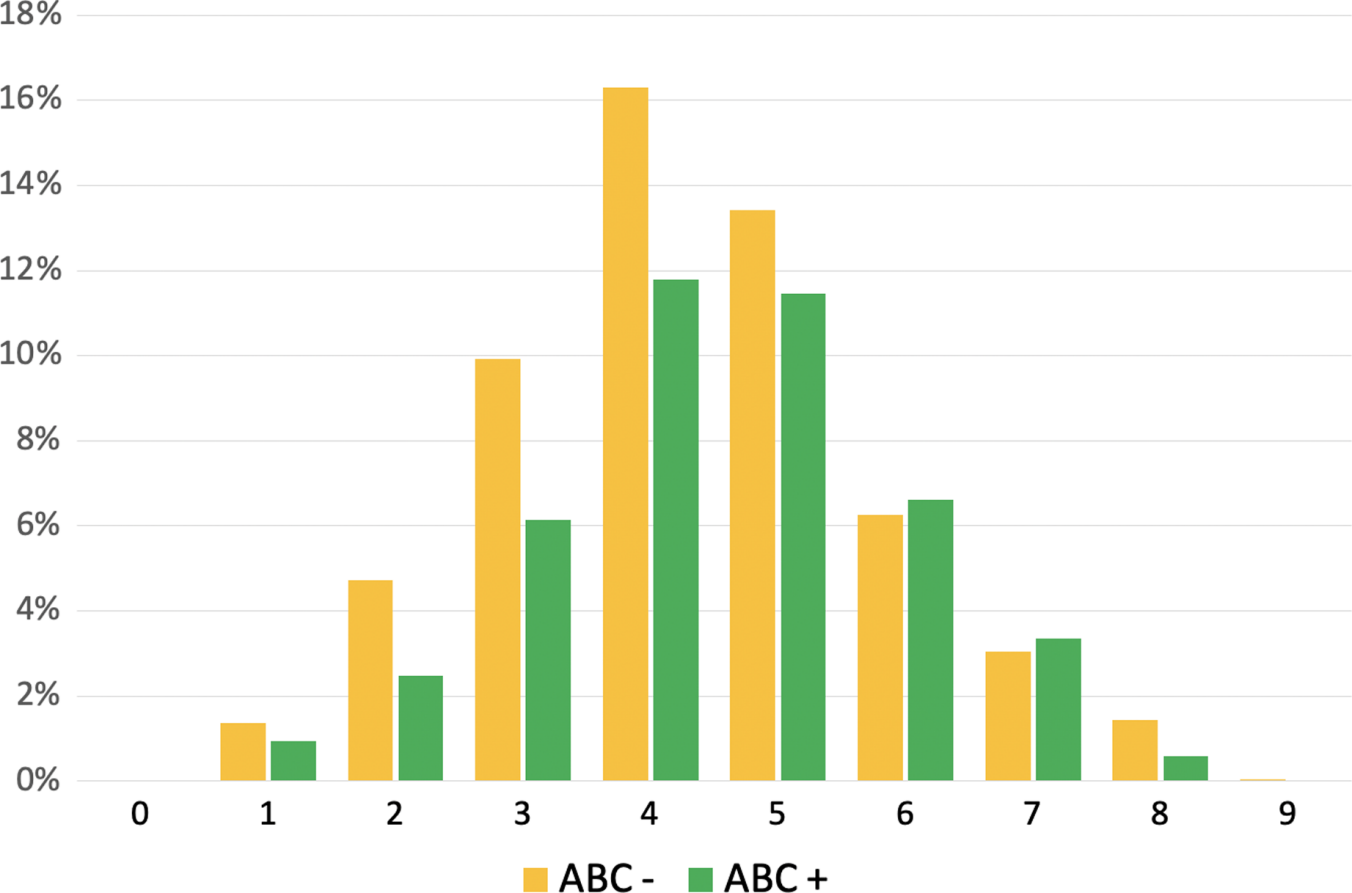
Distribution of cohort according with total 4S‐AF score and ABC adherence. ABC, atrial fibrillation better care

Among patients who were characterized using the 4S‐AF scheme, the median 4S‐AF score was 4 (IQR 3–5) in groups 2 and 5 (IQR 4–5) in group 3. The distribution of groups 2 and 3 based on total 4S‐AF score and ABC pathway compliance is presented in Figure 2. In group 2, 7/1795 (0.4%) patients fulfilled no ABC criteria, 222/1795 (12.4%) only 1 ABC criteria, 1232/1795 (68.6%) 2 ABC criteria and the remaining 334 (18.6%) patients fulfilled all 3 ABC criteria. In group 2, domain A non‐adherent patients were 1160/1461 (79.4%), domain B non‐adherent patients were 45/1461 (3.1%) and domain C non‐adherent patients were 492/1461 (33.7%). The distribution of patients according to the various domains of the 4S‐AF scheme is shown in Table S4.

### 3.1 4S‐AF scheme characterization and ABC pathway compliance on adverse outcomes

At 1‐year follow‐up, 513 patients reached the composite endpoint of all‐cause death/any TE event, 435 patients died, 102 had a TE event and 98 a major bleeding.

**FIGURE 3:**
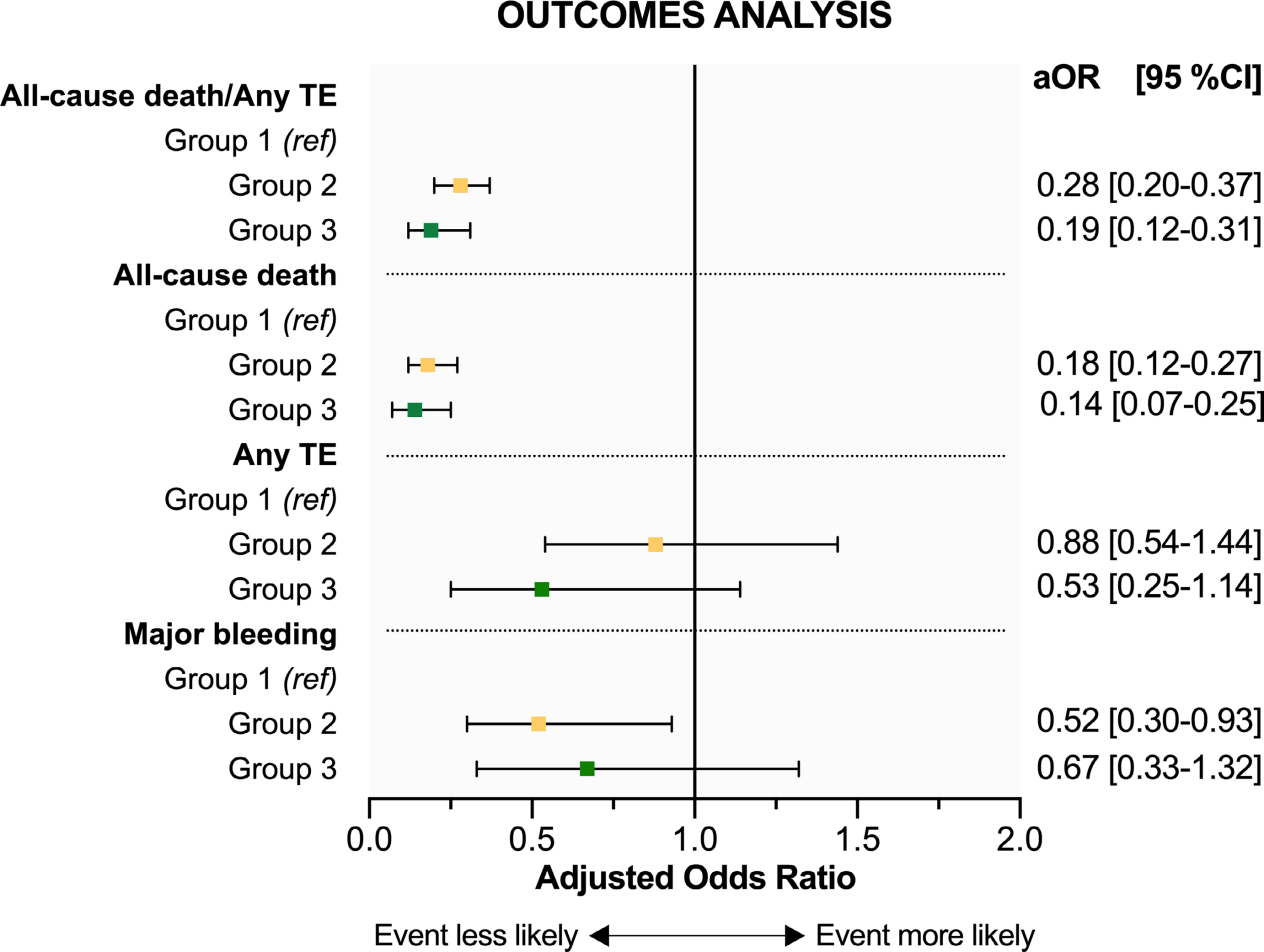
Forest plot showing the effects of 4S‐AF scheme characterization or ABC pathway compliance (group 2) and 4S‐AF scheme characterization plus ABC adherent management (group 3) on the composite outcome of all‐cause death/any thromboembolic event; all‐cause death; any thromboembolic event and major bleeding. Odds ratios are adjusted for age, gender, chronic kidney disease, chronic obstructive pulmonary disease, coronary artery disease, diabetes mellitus, heart failure, hypercholesterolaemia, hypertension and prior stroke. aOR, adjusted odds ratio; CI, confidence interval; TE, thromboembolism

On multivariable regression analysis (Figure 3), group 2 and group 3 were independently associated with reduced odds of the composite endpoint of all‐cause death/any TE event, with group 3 showing the highest advantage (OR: 0.19; 95% CI 0.12–0.31) [for group 2: OR: 0.28; 95% CI 0.20–0.37]. Similar results were observed for all‐cause death (group 2: OR: 0.18; 95% CI 0.12–0.27; group 3: OR: 0.14; 95% CI 0.07–0.25), but no significant association with TE events was evident (group 2: OR: 0.88; 95% CI 0.54–1.44; group 3: OR: 0.53; 95% CI 0.25–1.14). Only group 2 was associated with less odds of major bleeding (OR: 0.52; 95% CI 0.30–0.93). Among 1455 ABC compliant patients, 334 (23.0%) were not characterized using the 4S‐AF scheme. A low number of composite outcomes (*n* = 21) were observed in this subset of patients and 4S‐AF scheme characterization was not associated with reduced odds of the composite endpoint on the multivariable regression analysis (OR: 1.66; 95%CI 0.48–5.76).

## 4 DISCUSSION

Our study describes the effects of insufficient AF management according to the 2012 ESC guidelines^21^ and the potential benefits of an entirely ESC 2020 guideline‐directed management^3^ for AF patients, comprised of clinical characterization through the novel 4S‐AF scheme and ABC pathway adherent approach to integrated care, in a large contemporary cohort of Chinese patients. Our principal findings were as follows: (i) less than half of our cohort was characterized with the 4S‐AF scheme; (ii) less than one fifth of the cohort was characterized with the 4S‐AF scheme and treated accordingly with the ABC pathway for integrated care; (iii) appropriate patients’ characterization through the 4S‐AF scheme and ABC pathway adherent management were independently associated with better long‐term outcomes.

The 4S‐AF scheme and the ABC pathway have been recently implemented in the latest European guidelines on the management of AF^3^ and the 2021 Asia‐Pacific Heart Rhythm Society guidelines,^22^ but the clinical utility and prognostic value of these tools combined together has limited validation. Of note, no direct comparison between the 4S‐AF scheme and ABC pathway on clinical outcomes should be made because the former is meant to *characterize* AF patients while the latter to *guide patients’ treatment*. The 4S‐AF scheme incorporates four domains (Stroke risk, Symptom severity, Severity of AF burden and Substrate), evaluating clinically relevant aspects of AF management.^6^ We demonstrated that a structured and holistic approach including the 4S‐AF scheme or the ABC pathway was independently associated with a reduction of the composite endpoint (all‐cause death/any TE event) and all‐cause death. However, characterization of patients using the 4S‐AF scheme was fully done in only approximately 40% of the overall cohort and given its prognostic implications, appears to be well worth implementing. This comprehensive and structured evaluation of AF patients requires only routinely collected (and easy to obtain) clinical data. Its simplicity also allows the potential to identify high‐risk AF patients, facilitate communication among physicians and guide treatment decision‐making.^23^

Consequently, the ABC pathway provides a ‘step‐by‐step’ comprehensive approach to holistic and integrated management of AF patients.^7^ Its simplicity is particularly valuable as AF patients are often managed by different healthcare professionals (cardiologists, general practitioners and non‐cardiologists). The benefits of ABC pathway adherent care have been clearly shown to be associated with lower all‐cause death, cardiovascular death, stroke, major bleeding and cardiovascular events compared to usual care.^8^, ^9^, ^10^, ^11^, ^12^

Our data suggest that an ABC pathway adherent management on top of a careful 4S‐AF guided characterization of AF was independently associated with a reduction of the composite endpoint (all‐cause death/any TE event) and all‐cause death. Furthermore, there may be room for implementation of these new management strategies through mobile health technology, and indeed, the impact has been tested in the mAFA‐II trial, a prospective cluster randomized trial which showed a significant reduction in the composite outcome of stroke/TE, mortality, bleeding and hospitalizations, when compared to usual care.^24^, ^25^

These data reinforce the concept that underlying comorbidities, rather than the arrhythmia itself, determine prognosis in AF patients. Herein, a multidimensional AF characterization and a holistic, patient‐centred clinical management acknowledging the role of risk factors and multimorbidity, is associated with improved overall clinical outcomes.

### 4.1 Limitations

The main limitation of this study is related to potential misclassification bias due its observational nature. The use of continuous cardiac monitoring to determine the total AF burden was restricted, we were unable to assess LA fibrosis and LA diameter was used to quantify LA dilation instead of LA volume, which might be a more robust parameter for risk stratification.^26^ This survey was conducted before the publication of the current AF guidelines^3^ implementing the 4S‐AF scheme and ABC pathway into AF care and the enrolment period was relatively long. Therefore, changes in AF characterization strategies and clinical management cannot be completely ruled out. We could not exclude the possibility of residual confounders despite the multivariable analysis adjusting for a large set of covariates. Older age and a high burden of comorbidities may have led to undertreatment and worse prognosis. Further prospective studies, specifically designed and adequately powered, are needed to confirm our results.

## 5 CONCLUSIONS

In a contemporary real‐word cohort of Chinese AF patients, it is feasible to characterize and manage AF patients using the novel 4S‐AF scheme and ABC pathway for integrated care, respectively. The use of both these simple tools for AF patient characterization and holistic care is associated with improved clinical outcomes.

## CONFLICT OF INTEREST

GYHL: Consultant and speaker for BMS/Pfizer, Boehringer Ingelheim and Daiichi‐Sankyo. No fees are received personally. The other authors have no conflict of interest.

## AUTHORS' CONTRIBUTIONS

This paper has not been submitted for publication to any other journal. All authors have made a significant contribution and have read and approved the final draft. Y. Guo, J. Imberti and A. Kotalczyk contributed equally to design the study, interpret data and draft the manuscript (joint first authors); Y. Wang and GYH Lip contributed in the interpretation of data and revised the manuscript critically for important intellectual content (joint senior authors).

## Supporting information

Tables S1‐S4
